# 2160. Emergence of INDEL Mutations in KPC-2: Insights into Ceftazidime-Avibactam and Imipenem-Relebactam Efficacy

**DOI:** 10.1093/ofid/ofad500.1783

**Published:** 2023-11-27

**Authors:** Florencia Brunetti, Christopher R Bethel, María Belén Sanz, Fernando Pasterán, Gabriel Gutkind, Sonia Gomez, Robert A Bonomo, Pablo Power

**Affiliations:** Universidad de Buenos Aires, CABA, Ciudad Autonoma de Buenos Aires, Argentina; Louis Stokes Cleveland Department of Veterans Affairs Medical Center, Cleveland,, Cleveland, Ohio; ANLIS-MALBRÁN, CABA, Ciudad Autonoma de Buenos Aires, Argentina; ANLIS-MALBRÁN, CABA, Ciudad Autonoma de Buenos Aires, Argentina; Universidad de Buenos Aires, CABA, Ciudad Autonoma de Buenos Aires, Argentina; ANLIS-MALBRÁN, CABA, Ciudad Autonoma de Buenos Aires, Argentina; Louis Stokes Cleveland Department of Veterans Affairs Medical Center, Cleveland,, Cleveland, Ohio; Universidad de Buenos Aires, CABA, Ciudad Autonoma de Buenos Aires, Argentina

## Abstract

**Background:**

*Enterobacterales* producing KPC-2 and KPC-3 β-lactamases (Bla) are a significant clinical threat. Ceftazidime/avibactam (CZA) and imipenem/relebactam (IMI/REL) are successful in the treatment for these infections. Unfortunately, CZA-resistant isolates are emerging as a result of amino acid changes in three “hotspots” in the KPC Bla: Ω-loop region; the 237-243 loop; and the 266-275 loop (Figure 1). Our goal here is to assess the effect of insertion-deletion (INDEL) mutations located in the three hotspots in KPC-2, and their impact on the efficacy of these two drug combinations

**Figure:**
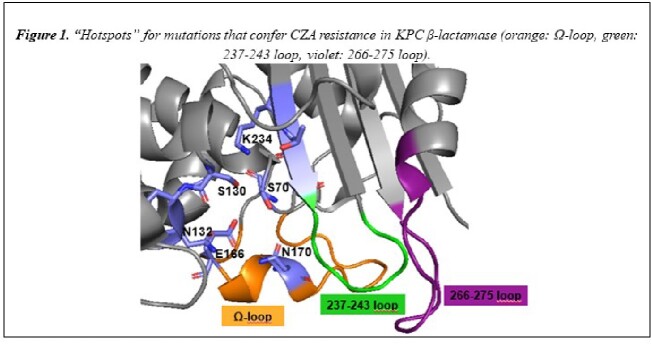

**Methods:**

The *bla*_KPC-44_ (ins_261_AVYTRAPNKDDKHSE), *bla*_KPC-80_ (ins_267_PNK), *bla*_KPC-81_ (del_173_I), *bla*_KPC-97_ (ins_276_VNSEA), and *bla*_KPC-160_ (del_167_LE) genes were amplified by PCR from *K. pneumoniae* isolates resistant to CZA. The *bla*_KPC-14_ (del_242-243_GT) gene was obtained by mutagenesis from the *bla*_KPC-2_ gene. Genes were cloned in pMBLe and transformed in *E. coli* TOP10 for MIC determinations. Immunoblotting with polyclonal anti-KPC-2 antibody was performed in whole-cell preparations. Molecular modeling of KPC variants were obtained by Yasara

**Results:**

All KPC variants demonstrated lower MICs compared to KPC-2 for most β-lactams (carbapenems included), except for CAZ, where variants exhibited up to 8-fold higher MICs. All variant clones showed at least a 3-dilution higher MICs for CZA, except KPC-160. IMI-REL restored susceptibility to KPC-2 and INDEL variants. The Ω−loop variants exhibited lower MICs compared to the other hotspots variants (CZA included). INDEL mutations resulted in decreased levels of protein production, with KPC-81 production being the most affected. *In silico* models suggest that INDEL mutations have different effects depending on the structural domain affected

**Table 1.**
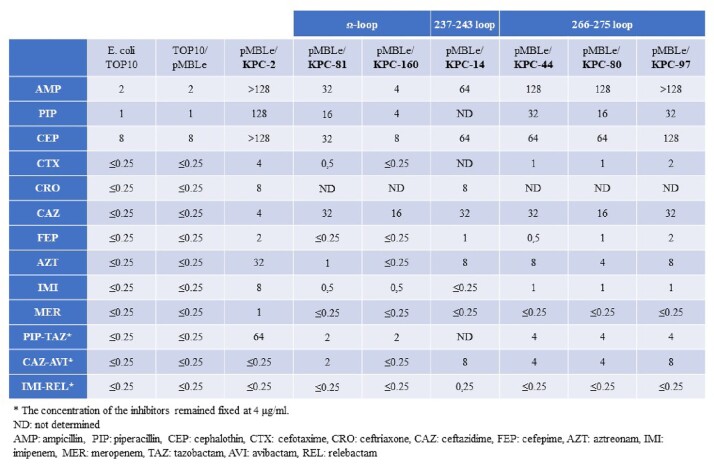
Minimum Inhibitory Concentrations (μg/mL) of E. coli TOP10F´ recombinant clones

**Figure:**
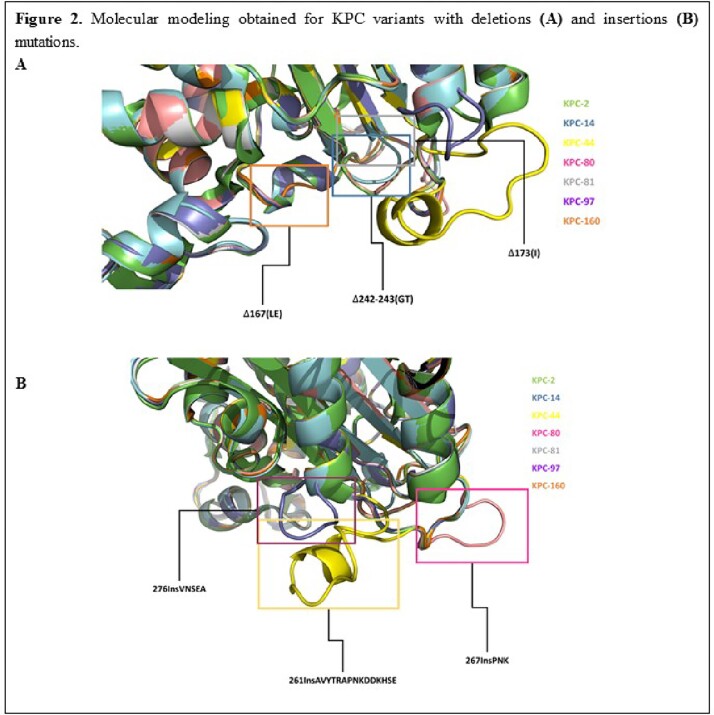

**Conclusion:**

INDEL mutations in the three hotspots of KPC-2 can impair CZA activity, but not affect IMI/REL susceptibility. The differences in MICs among the variants suggest that each hotspot affects the interactions of KPC-2 with CAZ and AVI through different mechanisms. Additionally, INDEL mutations appear to decrease protein stability as Bla production levels are reduced. This work provides relevant information about the changes in the interaction with DBO inhibitors associated with INDEL mutations in KPC-2

**Disclosures:**

**Robert A. bonomo, MD**, Entasis, Merck, VenatoRx, Wockhardt: Grant/Research Support

